# Genetic Diversity of *Trypanosoma cruzi* in Panama Inferred by Multi-locus Sequence Typing of Mitochondrial Genes

**DOI:** 10.3390/microorganisms10020287

**Published:** 2022-01-26

**Authors:** Jose E. Calzada, Franklyn Samudio, Corina de Juncá, Vanessa Pineda, Barbara A. Burleigh, Azael Saldaña

**Affiliations:** 1 Departamento de Investigación en Parasitología, Instituto Conmemorativo Gorgas de Estudios de la Salud, Panama 0816, Panama; jcalzada@gorgas.gob.pa (J.E.C.); fsamudio@gorgas.gob.pa (F.S.); vpineda@gorgas.gob.pa (V.P.); 2 Facultad de Medicina Veterinaria, Universidad de Panamá, Apartado 3366, Panama; 3 Departamento de Genética y Biología Molecular, Facultad de Ciencias Naturales, Exactas y Tecnología, Universidad de Panamá, Apartado 3366, Panama; 4 Centro de Investigación y Diagnóstico de Enfermedades Parasitarias (CIDEP), Facultad de Medicina, Universidad de Panamá, Apartado 3366, Panama; juncacorina@yahoo.com; 5 Department of Immunology and Infectious Diseases, Harvard School of Public Health, 655 Huntington Ave, Boston, MA 02115, USA

**Keywords:** Chagas disease, *Trypanosoma cruzi*, genetic diversity, discrete typing units, Panama, Central America

## Abstract

The objective of this study was to provide information on *Trypanosoma cruzi* genetic diversity among isolates obtained from different biological sources circulating in endemic areas of Panama. Initial discrete typing units (DTUs) assignment was performed evaluating three single locus molecular markers (mini-exon, heat shock protein 60 and glucose-6-phosphate isomerase genes). Further diversity within TcI lineages was explored using a multi-locus sequence typing approach with six maxicircle genes. Haplotype network analysis and evolutionary divergency estimations were conducted to investigate the genetic relatedness between Panamanian TcI isolates and isolates from different endemic regions in the Americas. Our molecular approach validated that TcI is the predominant DTU circulating in Panama across different hosts and vector species, but also confirmed the presence of TcIII and TcVI circulating in the country. The phylogenetic tree topography for most Panamanian TcI isolates displayed a high level of genetic homogeneity between them. The haplotype network analysis inferred a higher genetic diversity within Panamanian TcI isolates, displaying eight different haplotypes circulating in endemic regions of the country, and revealed geographical structuring among TcI from different endemic regions in the Americas. This study adds novelty on the genetic diversity of *T. cruzi* circulating in Panama and complements regional phylogeographic studies regarding intra-TcI variations.

## 1. Introduction

Chagas disease, caused by *Trypanosoma cruzi,* is a major neglected health problem in Central America, leading to significant economic losses due to reduced productivity from early-age disability and mortality [1]. More than 10% of people in this region live in Chagas disease risk areas, and nearly 400,000 people are estimated to be infected with this protozoan parasite [2,3,4].

**Figure 1:**
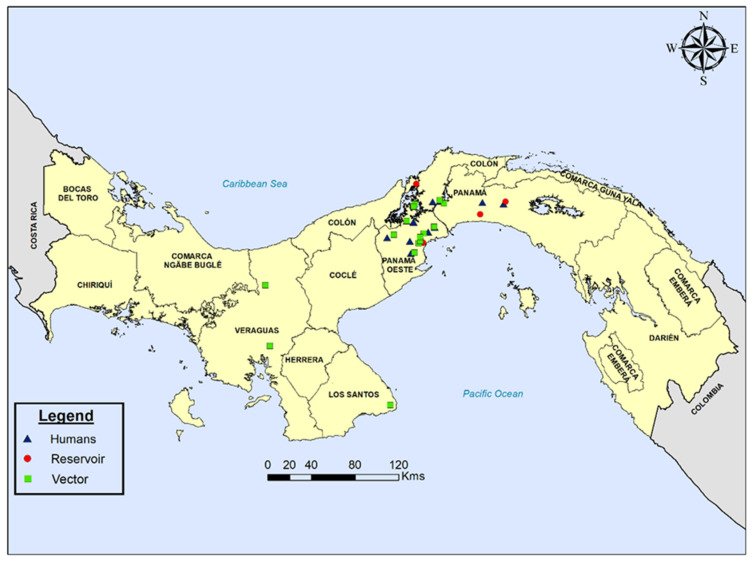
Map of Panama showing the geographical origin and biological source of the 32 Panamanian *Trypanosoma cruzi* isolates analyzed in this study. Blue triangles represent samples isolated from humans, red circles from reservoirs and green squares from vectors.

In Panama, situated in the southernmost part of Central America (Figure 1), Chagas disease seems to adopt clinical and epidemiological features that may differ from the rest of the region. For instance, the main Chagas disease vector in Panama is *Rhodnius pallescens*; a sylvatic triatomine closely associated with “royal” palm trees (Attalea butyracea) [5,6,7,8], differing from the main vector species in the rest of the Central American region. *Rhodnius pallescens* is also the only known vector of *Trypanosoma rangeli*, a non-pathogenic trypanosome that is transmitted through the saliva of this triatomine [6,9,10,11,12] and which is frequently found in blood of both humans and animals in Panama. Interestingly, in animal models, it has been observed that exposure to *T. rangeli* may modulate immune responses that confer some degree of protection against a subsequent infection with *T. cruzi* [13,14,15,16].

Some clinical aspects of Chagas disease in Panama also differ from those documented in South America and, to some extent, to other Central American regions. For example, Chagoma and Romaña’s signs during the acute phases of infection are rarely reported [17,18] and parasitemia in humans is low and of relatively short duration compared to acute infections documented in South America [18,19]. Moreover, symptomatic Chagas disease appears to be milder and is often characterized as the cardiotropic form with no evidence of the gastrointestinal manifestations of developing in the chronic stages of infection [18,19,20]. It has been suggested that these clinical differences might be related to the genetic variability of *T. cruzi* parasites circulating in Panama and/or to the immunogenetic background of host populations [8]. However, there are no recent studies that address the pathogenesis and clinical manifestations of Chagas disease observed in the country. Furthermore, the genetic structure of *T. cruzi* circulating in the different endemic regions of Panama has only been partially investigated, and only at the most basic level of discrete typing units (DTUs) using single locus markers [21,22,23,24,25].

At present, seven DTUs of *T. cruzi* (TcI–TcVI and Tcbat) are internationally accepted [26,27,28]. Within each of these groups there is substantial genetic heterogeneity, TcI being the most diverse and widespread in humans, vectors and reservoirs [29,30], as well as the predominant lineage in the Central American region [3,28]. It has been suggested that the geographical heterogeneity observed in Chagas disease pathology, biological behavior, transmission cycles and clinical outcome might be related to genetic variability displayed by this parasite [31,32]. Thus, genotype identification is important for epidemiologic tracking purposes, and as a guide to provide a prognosis of disease progression and/or differential treatment decisions based on the infective strain.

In this regard, few studies have explored the genetic diversity of *T. cruzi* strains circulating in endemic regions of Panama by surveying human hosts, vectors and animal reservoirs in these regions to understand the epidemiologic importance of genotypes in transmission cycles and disease. Earlier studies have identified TcI as the predominant DTU circulating in Panama, and in many cases associated with disease [21,22,23,24,25]. However, these studies were limited by the low number of samples analyzed from different biological samples (human, vector, animal), the restricted geographical range, the lack of standardization of molecular protocols, as well as the short time span over which samples were collected.

To address this gap, the objective of present study was to provide information on *T. cruzi* discrete typing unit genotypes in isolates obtained from different biological sources circulating in endemic areas of Panama. We also explored the genetic diversity within TcI lineages based on a maxicircle multi-locus sequence typing approach (mtMLST). Haplotype network analysis and evolutionary divergency estimations between mitochondrial sequences were further conducted to investigate the genetic relativeness between Panamanian TcI isolates and isolates from different endemic regions in the Americas.

## 2. Materials and Methods

Panama is a narrow and highly biodiverse biogeographical corridor connecting South and Central America (Figure 1). It has a tropical climate, with relatively high temperatures throughout the year, ranging from 26 °C to 32 °C, and two marked seasons: Dry Season: January–May; Rainy Season: May–December. The Pan American Health Organization (PAHO) considers Panama an area endemic for Chagas disease where the sylvatic behavior of the main vectors recognized in the country (*R. pallescens*, *Triatoma dimidiata* and *Panstrongylus geniculatus*) complicates current approaches to control vectorial transmission [4,33]. These triatomine species are widespread and are responsible for maintaining sylvatic transmission cycles that vary in intensity across different rural and suburban regions of Panama. Compared to other countries from the region, human infections in endemic areas of Panama are low (prevalence ranging 1–4%) and are mostly associated with sporadic invasion by sylvatic vectors of vulnerable poor-quality housing predominant in rural areas of the country [8,9,10,11,12,34]. Several sylvatic and domestic animal reservoirs have been identified in the country, from which opossums (*Didelphis marsupialis*) are the most important in the transmission cycle [18]. They share the same natural habitat with vectors (royal palm trees) and due to their synanthropic behavior, opossums favor the connection between sylvatic and peridomestic *T. cruzi* cycles, particularly in deforested and forest fragmented rural areas of Panama, where there is an increase in vector abundance and increased risk of human infection by *T. cruzi* [35,36].

### 2.1. Sample Collection and T. cruzi Genotyping

A panel of 32 Panamanian parasite isolates obtained from different epidemiological settings were genotyped (12 from the blood of Panamanian patients with different clinical profiles, 4 from the blood of animal reservoirs and 16 from vectors). Information regarding the clinical case, identification of the isolates, location, and year of isolation is presented in Figure 1 and Table 1. The Panamanian isolates were supplied by the Trypanosomatids Bank at CIDEP, Faculty of Medicine, University of Panama and were maintained as epimastigotes in liver infusion-tryptose (LIT) medium supplemented with 15% fetal bovine serum. Reference strains used in this study were obtained from the Department of Immunology and Infectious Diseases, Harvard School of Public Health. Total genomic DNA was prepared from logarithmic phase cultures using Wizard^®^ Genomic DNA Purification System (Promega, Madison, WI, USA).

**Figure 2:**
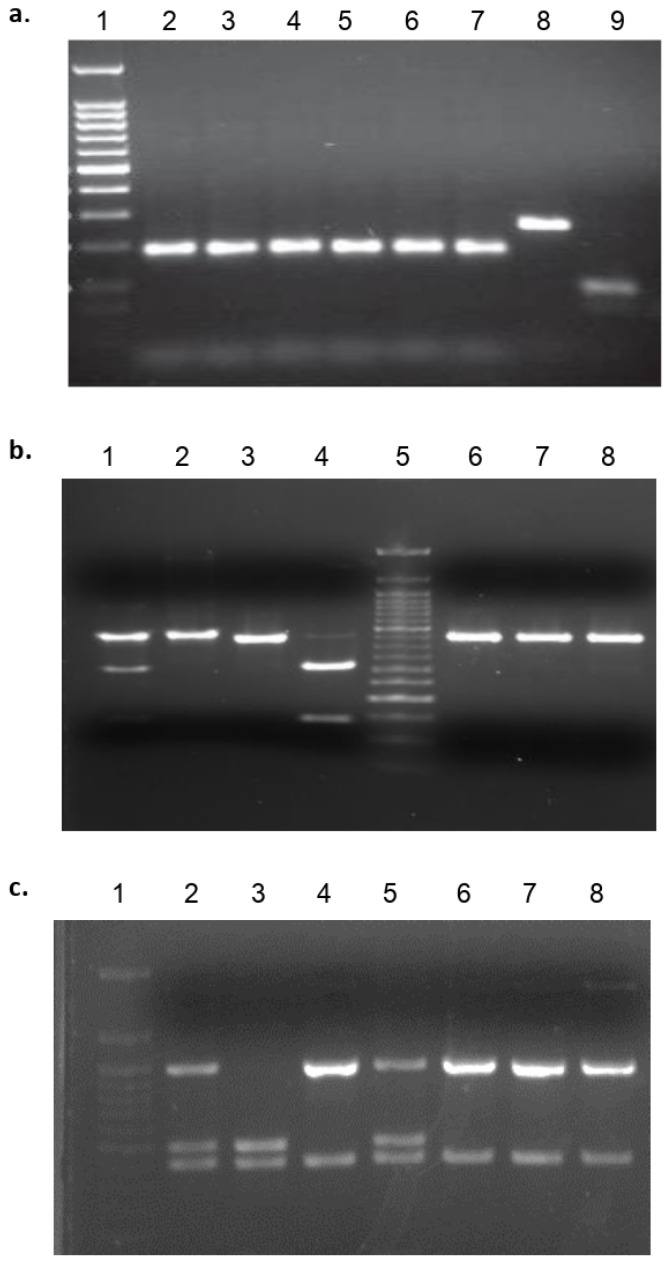
*Trypanosoma cruzi* genotyping. Initial discrete typing unit (DTU) assignment was performed evaluating the following single locus molecular: mini-exon gene, heat shock protein 60 (HSP60)/EcoRV and glucose-6-phosphate isomerase (GPI)/HhaI: (**a**) Representative agarose gel for *Trypanosoma cruzi* mini-exon genotyping. Lane 1: Molecular weight marker 50-bp ladder; Lanes: 2–7: *T. cruzi* TcI Panamanian isolates; Lane 8: *T. cruzi non*- TcI Panamanian isolate; Lane 9: *T. rangeli* control; (**b**) Representative agarose gel for *Trypanosoma cruzi* HSP60/EcoRV genotyping. Lanes 1–3: T. cruzi control strains; Lane 4: TCH1; Lane 5: Molecular weight marker 50-bp ladder; Lane 6: TCH2; Lane 7: TCH3; Lane 8: TCH4; (**c**) Representative agarose gel for *Trypanosoma cruzi* GPI/Hha1 genotyping. Lane 1: Molecular weight marker 50-bp ladder; Lanes 2–4: *T. cruzi* control strains; Lane 5: TCH1; Lane 6: TCH2; Lane 7: TCH3; Lane 8: TCH4.

Genotyping was accomplished in a two-stage approach. Initial DTU assignment was performed evaluating the following single locus molecular markers as previously recommended [37]: mini-exon gene, heat shock protein 60 (HSP60)/*EcoR*V and glucose-6-phosphate isomerase (GPI)/*Hha*I. All mixtures, amplification conditions and restriction enzyme digestions were previously described [37]. Amplification and RFLP products were visualized on 1.5% agarose gels containing ethidium bromide, and their sizes were estimated by visual inspection using a 50 bp DNA ladder as molecular size marker (New England Biolabs, Ipswich, MA, USA) (Figure 2).

To further explore intra-DTU diversity within Panamanian isolates assigned as TcI, six maxicircle gene fragments were amplified: ND1 (NADH dehydrogenase subunit 1), COII (cytochrome c oxidase subunit II), MURF1 (Maxicircle unidentified reading frame 1, CYT b (cytochrome b), 12S rRNA and 9S rRNA, coding regions. Primers, annealing temperatures and PCR amplification conditions were as previously described [30]. As controls, we also sequenced the following reference *T. cruzi* TcI strains: Y strain (TcII, isolated from a patient in Sao Paolo, Brazil), Tulahuén strain (TcVI, isolated from *Triatoma infestans* collected in Tulahuén, Chile), CL Brener strain (TcVI, isolated from *Triatoma infestans* in Rio Grande Do Sul, Brazil), G strain (TcI, isolated from a naturally infected opossum in Amazon, Brazil) and a recent TcI isolate from an El Salvadorian patient [38].

PCR amplification products were separated on 1.0% agarose gels prepared with 1X TBE buffer. Resulting DNA bands were excised from agarose gel and purified using the Qiaquick gel extraction Kit (Qiagen, CA, USA) following the manufacturer’s instructions. Purified PCR products were sequenced in both directions at the GENEWIZ DNA Sanger Sequencing Service using previously described primers [30]. Nucleotide sequences for all six gene fragments are available in the GenBank database under the accession numbers listed in Table S1.

### 2.2. Sequence Editing and Bayesian Phylogenetic Inference

Chromatograms were edited by the assembling-to-reference tool of UGENE toolkit using a trimming quality value of 35 [39]. For each isolate, maxicircle sequences were concatenated according to their structural arrangement. Multiple sequence alignments were generated by MAFFT software also included in the bioinformatic toolkit UGENE with a maximum number of iterative refinements of 3 and a gap penalty of 1.53. Using JModelTest 2 [40], the best DNA evolution model was found to be the GTR (GTR + I + G) model. A phylogenetic tree reconstruction of *T. cruzi* was implemented applying Bayesian inference (BI) with the Mr. Bayes v.3.2 program. Ten Markov chains were performed for 8 million generations, and trees were sampled for every 1000 generations. Twenty-five percent of the sample trees were discarded, and the remaining trees were used to build up a consensus tree and calculation of posterior probabilities of clades. The result of Bayesian analyses was visualized using Figtree v1.4.2. *T. rangeli* concatenated sequences were used as root. Transformation of the leaves and schematically representation of the root were applied for visualization purposes.

### 2.3. Haplotype Network Construction

The Network 5.0 software [41] was used to construct all possible shortest least complex phylogenetic trees from concatenated sequence of both *T. cruzi* reference strains and Panamanian isolates using the median joining algorithm that permit multi-state data and setting the epsilon parameter of the algorithm. Additionally, we use the MP algorithm of Network software to get rid of all unnecessary median vectors and the star contraction option to look for any demographic expansion defining different color-coded nodes for each country of origin of the *T. cruzi* strains/isolates used herein.

### 2.4. Estimation of Evolutionary Divergency between TcI Mitochondrial Sequences

To assess the evolutionary divergence between *T. cruzi* TcI sequences, we initially calculated the *p*-distance and the number of base differences per sequence from between sequences using the evolutionary software Mega X [42]. Sixty-nine (29 from this study and 40 from reference strains) concatenated sequences were used to do the analysis including coding positions 1st + 2nd + 3rd and noncoding sites. All ambiguous positions were removed for each of the sequence pairs. There were a total of 2284 positions in the final dataset. Based on the *p*-distance and sequence similarity results, we grouped TcI haplotypes in five major geographical groups named: Brazil A, Brazil B, Brazil C, Andean countries, North-Central American and Western South American countries (NCWS). After grouping concatenated sequences, we estimated the evolutionary divergency between groups by assessing base differences per site from averaging over all sequence pairs between groups. To assess these differences, we computed differences between group mean distance by obtaining the *p*-distances and the number of differences between groups. The analyses were performed in 69 sequences including coding positions 1st + 2nd + 3rd and noncoding sites. After removing ambiguous positions, 2284 positions remained in the final dataset.3.

## 3. Results

**Figure 3:**
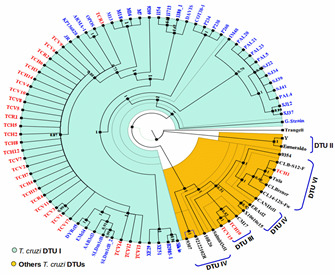
Phylogenetic tree inferred from Bayesian analysis showing relationships between 32 *Trypanosoma cruzi* isolates from Panama (in red color) and 55 reference *T. cruzi* strains across the Americas. TcI reference isolates are in blue color. The tree was constructed with the MrBayes v.3.2 program using concatenated mitochondrial sequences of six *T. cruzi* loci (ND1, COII, MURF1, CYT b, 12S rRNA and 9S rRNA). Ten Markov chains were proceeded for 8 million generations, and trees were sampled for every 1000 generations. *Trypanosoma rangeli* concatenated sequences were used as root.

We retrospectively genotyped 32 *T. cruzi* Panamanian isolates: 12 from humans with different clinical profiles, four from reservoirs and 16 from vectors collected in different endemic regions of Panama between 1965 and 2016 (Figure 1 and Table 1). Initial molecular analysis using the triple marker (mini-exon, HSP60 and GPI markers) confirmed that DTU TcI was the predominant *T. cruzi* genotype (90.3%; 28/31) found across all sources and time span. However, two human isolates from acute human cases (TCH1 and TCH10) and one (TCV15) from a secondary vector (*P. geniculatus*) displayed a non-TcI profile using this molecular approach. The mitochondrial MLST analysis further confirmed that TCH10 and TCV15 belong to the TcIII, and TCH1 to the TcVI DTUs (Figure 3).

### 3.1. Phylogenetic Tree and Haplotype Network Results

Reconstruction of a phylogenetic trees was conducted using the concatenated mitochondrial sequences of six loci (ND1, COII, MURF1, CYT b, 12S rRNA and 9S rRNA) from 87 *T. cruzi* isolates, including the 32 from this study along with 55 reference isolates retrieved from the GenBank database (Figure 3). Complete sequence alignment of the concatenated genes from this study is available upon request. The inferred phylogenetic tree based on Bayesian analysis showed a distinct clustering among the different *T. cruzi* DTUs analyzed. Most Panamanian samples (29/32) grouped within the TcI cluster. Confirming the triple marker initial results, only three samples from this study (TCH1, TCH10 and TCV15) grouped outside the TcI cluster: TCH10 and TCV15 clustered with reference samples belonging to the TcIII DTU, and TCH1 with reference samples from the TcVI DTU (Figure 3).

Among the 29 TcI Panamanian isolates, the majority were genetically homogenous and grouped together in a well-defined clade (Figure 3). However, two isolates from secondary vectors (TCV1 and TCV3) and three samples from the main Panamanian vector (TCV12, TCV13 and TCV14) showed some degree of genetic variability grouping in subclades within the TcI main Panamanian cluster, more closely related to reference isolates from Colombia. Interestingly, samples TCV12 and TCV13 originated from insects that were collected recently from non-traditional endemic regions located in the western side of Panama (Figure 1 and Table 1). Furthermore, sample TC3 that was isolated from an otter (*Lontra longicaudis*) in 1973, displayed a higher level of diversity clustering separately with TcI reference strains (OPOS and ARMA-1) isolated from opossums in southern USA.

**Figure 4:**
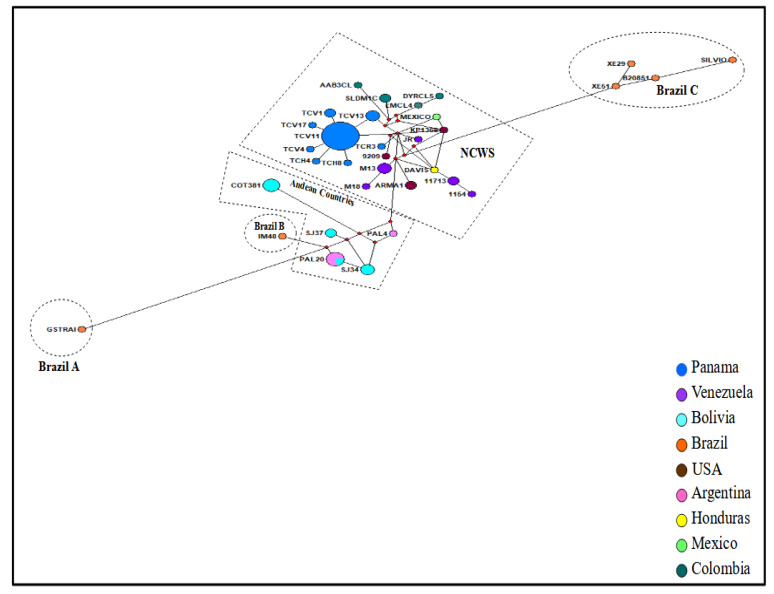
Haplotype network inferred by a median-joining method using concatenated mitochondrial sequences of six loci (ND1, COII, MURF1, CYT b, 12S rRNA and 9S rRNA) from 69 *Trypanosoma cruzi* TcI isolates, 29 from Panama (blue circles) and 40 reference *T. cruzi* strains from different endemic countries across the Americas. Circles represent a unique sequence haplotype with the color denoting the country origin and the size of the circle accounting for its frequency. The lengths of the lines connecting the haplotypes refer to the distance of relatedness. The small red circle represents the median vector, which can be interpreted as an unsampled sequence or an extinct ancestral sequence. The five clusters (Brazil A, Brazil B, Andean Countries, NCWS and Brazil C) are enclosed within dashed lines.

Results from the haplotype network analysis among the concatenated TcI sequences (29 from this study and 40 reference isolates from different Chagas endemic countries), revealed the presence of 33 different haplotypes (Figure 4 and Table S2). Based on the nucleotide diversity and genetic distance observed among the haplotypes, five different regional groups (clusters) were evidenced: Andean region, Brazil A, Brazil B, Brazil C, and North and Central American Region—Western South American Countries (NCWS) (Figure 4, Tables S2–S4). As expected, due to its geographical proximity, Panamanian TcI isolates were positioned within this last regional group (NCWS), displaying eight different haplotypes (Haplotypes 18–25) circulating in endemic regions of the country (Figure 4 and Table S2). The Panamanian haplotypes (blue circles) formed a cluster of nodes (*n* = 6) surrounding a major node (Haplotype 18-TCV11) conformed by 19 identical sequences. This haplotype was the most frequent (19/29; 65.5%) and widespread among the Panamanian isolates across the different regions and biological origin of the isolates. The other Panamanian haplotypes were less frequently detected (between 1 and 3 samples for each haplotype) and showed high relatedness with haplotype 18-TCV11, from which, most probably, they expanded. Consistent with the phylogenetic tree results, haplotype 25-TCR3 was genetically more distant from the major Panamanian haplotype cluster, and closer to haplotypes from Venezuela. It was represented by only one sample that originated from an otter; a wild animal not considered a common reservoir for Chagas disease. In general, Panamanian samples seem to have a distinct geographical position, more closely related to isolates from northern South America (particularly Colombia) and from Central America. As expected, samples from the most southern countries of Brazil, Bolivia and Argentina were more distantly related from the Panamanian samples.

### 3.2. Nucleotide Divergence between Panamanian TcI Isolates and Reference Strains

The calculation of *p*-distance and the number of base differences between mitochondrial concatenated sequences showed that the TcI Panamanian isolates were mostly homogenous. Twenty-six isolates out of 28 Panamanian isolates had *p*-distance value of zero and showed no nucleotide differences along the concatenated sequences after removing ambiguous positions for the analysis. However, isolates TCV17 and TCR3 were more divergent showing *p*-distance values of 0.002 and 0.001, and nucleotides’ differences of four and two nucleotides, respectively (Tables S4 and S5).

The divergency analysis also revealed differences between Panamanian isolates and reference strains used in this study. These genetic differences were more evident between haplotype sequences that seem to be more genetically divergent (Figure 4). For this reason, we decided to group the sequences based on the grade of divergency between them. As a result, five groups containing haplotypes with a high level of sequence similarity and being geographically related were found. The groups encompass haplotypes from Brazil (groups Brazil A, Brazil B and Brazil C), Andean countries (Andean countries group) and haplotypes from North and Central American countries along with Western South American Countries (NCWS group).

All haplogroups showed different levels of divergence with respect to the Panamanian TcI isolates (Tables S3–S5). The group Brazil A, consisting only of the G-strain, was the most divergent showing nucleotide difference between 44 to 48 nucleotides and *p*-distance values between 0.04 to 0.019. Furthermore, Panamanian TcI isolates showed nucleotide differences from 22 to 33 nucleotides and *p*-distances varied from 0.10 to 0.15 compared with the Brazil C haplogroup that consisted of four isolates from Brazil, including the Silvio strain. The strain IM48, the only member of Brazil B group, showed less differences with the Panamanian isolates than other Brazilian haplogroups. This strain had 22–27 nucleotide differences and *p*-distance values of 0.010 to 0.012 when compared to the Panamanian isolates. On the other hand, the Andean countries group was found to be less divergent than the Brazilians haplogroups when compared with Panamanian isolate sequences. This Andean haplogroup had nucleotide differences of 13 to 24 nucleotides along concatenated sequences and *p*-distance values between 0.006 to 0.012. As expected, due to the geographic proximity, Panamanian isolates were more related to other members from the NCWS haplogroup which included isolates from North and Central America as well as isolates from Northern South America. Minor nucleotide differences and *p*-distance values were observed when compared with mitochondrial concatenated sequences inside this group. Nucleotide differences between 2 to 13 nucleotides and *p*-distance values of 0.001 to 0.006 were found between Panamanian isolates and reference strains belonging to this group.

## 4. Discussion

Since its formation about three million years ago, the Isthmus of Panama has been a biological corridor between North and South America for the interchange of reservoirs, vectors and the pathogens they have harbored (Figure 1), greatly shaping today’s biological diversity observed in the country [43]. In this context, *T. cruzi* ancestors can be traced to approximately 5–7 million years ago when it separated from *T. cruzi marinkellei* [44,45]. Between 1–3 million years ago, coinciding with the formation of the land bridge joining South and North America 3.5–3.1 MYA [43], this *T. cruzi* ancestor further diversified to form the 7 major groups (DTUs) recognized today. Thus, the strategic position of Panama makes it an ideal site for conducting studies on the genetic composition and population structure of *T. cruzi*. The potential association between parasite genetic diversity and ecobiological, pathogenic and epidemiological characteristics of Chagas disease [31,32,46,47], further emphasizes the relevance of this analysis. Moreover, *T. cruzi* genetic diversity has the potential to confound the performance of serologic diagnosis [31,47]. It has recently emerged that certain haplotypes circulating in North and Central America are associated with negative or inconclusive serological results, despite clear molecular evidence for infection. This is most likely due to the antigenic diversity exhibited by different *T. cruzi* strains [47,48]. Issues with interpretation of serodiagnostic tests are further complicated in countries like Panama where other *Trypanosomatidae*, such as *Leishmania* spp. and the non-pathogenic *T. rangeli*, coincide with *T. cruzi* in common endemic areas [10,18]. Indeed, cross-reactivity between these divergent parasites has been described, particularly in individuals coinfected with *Leishmania* spp. [49].

Our exploration of the genetic diversity of Panamanian *T. cruzi* isolates derived from different biological samples over a span of 57 years (1960–2017) confirmed that TcI was the predominant DTU circulating in the country in strong agreement with previous studies conducted in Panama [21,22,23,24,25]. TcI was found across all biological samples, including patients with different clinical profiles and a fatal acute infection in a dog (Table 1), reinforcing the current view that TcI occurs in both domestic and sylvatic cycles in nature and may exhibit an important degree of pathogenicity [46]. Geographically, TcI is the predominant DTU in the Americas, dispersed widely from southern USA to northern Argentina. This DTU is found throughout the range of triatomine distribution infecting many different mammal hosts, including humans [28,32]. Moreover, TcI infections are common in patients with different clinical outcomes, including cardiomyopathy and death, from the Amazon basin [46,50], a region where Chagas’ disease ecoepidemiology is similar to the one observed in endemic regions from Central Panama, especially as palms are hotspots for transmission between reservoir and triatomines.

Using our molecular genotyping approaches, it was also possible to unambiguously detect three isolates belonging to non-TcI genotypes: one TcVI and two TcIII. The TcVI DTU was detected in a patient from the central region of the country with an initial acute infection and a chronic evolution. In a recent whole-genome sequencing analysis of TcI isolates from six American countries, this TcVI isolate was mistakenly included as a TcI sample, and together with the highly virulent TcI Colombiana, was classified as an outlier [51]. In agreement with our results, a recent study assessing *T. cruzi* diversity through comparative genomics correctly assigned this isolate as TcVI [47]. Besides these findings, in the Mesoamerican region, TcVI has been previously described as infecting local vectors and humans from Mexico and Honduras [48,52,53,54], suggesting this DTU might be widely present, yet not frequently detected, across Mesoamerica. In the neighboring country of Colombia, TcVI has also been detected as infecting vector and human samples [55,56], with a very low prevalence [57]. In general, TcVI has mainly been associated with domestic transmission cycles in the Southern cone of South America, where severe forms of Chagas disease and congenital transmission are frequently reported.

This study also identified TcIII in a Chagasic patient and in a vector species (*P. geniculatus*). TcIII infections have been mainly associated with terrestrial ecotopes and *P. geniculatus* [58,59], which is a sylvatic vector that generally inhabits various vertebrate nests, especially the burrows of armadillos (*Dasypodidae*) and anteaters (*Myrmecophagidae*) [59], both common reservoirs found in the study area where the sample was isolated [18]. In Panama, *P. geniculatus* has also been reported with relative frequency to visit human dwellings located adjacent to forested areas, and the species has repeatedly been found infected with *T. cruzi* [8,18,60]. Although not frequently detected in humans, recent reports from Brazil and neighboring Colombia have described TcIII circulating in Chagasic patients with differing clinical forms [55,61]. We did not find TcIV in this study, although it is the most common secondary DTU reported in Central/North America [3].

The presence of non-TcI genotypes circulating in Panama was recently reported in a study conducted in a rural area from the eastern region of Panama where mixed infections (consisting of TcI coinfected with either TcII, TcV or TcVI) were found in chronic patients [25]. The molecular strategy employed in that study only permitted distinction between TcI and TcII/V/VI DTU groups. Unfortunately, we do not have isolates from this region to compare and confirm these interesting findings or to determine the exact DTUs implicated in the mix infections reported.

With few exceptions, the phylogenetic tree topography for most Panamanian TcI isolates displayed a high level of genetic homogeneity. One of the most divergent was TC3 that was isolated from an otter bled by one of our team members. It is difficult to speculate how this sylvatic semi-aquatic mammal that inhabits rivers in Panama became infected with *T. cruzi*. However, it can be argued that even though otters mainly feed on fish and crustaceans [62], the infection could have occurred via the ingestion of infected rodents or triatomine bugs present in their terrestrial burrows. Our network analysis was capable to capture a higher genetic diversity within Panamanian TcI isolates, displaying eight different haplotypes circulating in endemic regions of the country. Consistent with previous studies [3,29,47,51], the haplotype network also inferred a phylogeographic structuring among TcI isolates from different endemic regions. Inside this network, Panamanian TcI haplotypes clustered with the NCWS group that encompasses haplotypes belonging to geographical regions from North and Central America as well as haplotypes from the northernmost countries in South American (Colombia and Venezuela). Furthermore, results from the divergency analysis support this relationship, demonstrating that Panamanian TcI haplotypes are more genetically related to haplotypes from the NCWS group. It has been speculated that the tribe *Rhodniini* spread from the Amazon basin, passing through the Andes-Sierra Nevada to reach Colombia and then Central America, after the formation of the Panamanian isthmus around 2–4 MYA [63]. This might have opened the door to more recent speciation events in the genus *Rhodnius* in North and Central America regions, considering that the splits of *Triatomines* into *Rhodnius* and *Triatoma* occurred before the arrival of bats and rodents to South America [58]. TcI is mainly associated with the genus *Rhodnius* and shows a more generalized behavior regarding mammal host preferences. Therefore, the emergence of new *Rhodnius* species with diverse feeding patterns inhabiting sympatrically and/or allopatrically specific landscapes in Central and North America might have shaped the genetic structure of TcI in this region, as it has been suggested for *T. rangeli* [64,65,66]. The low genetic divergency found in TcI haplotypes from the NCWS group might be the result of recent vector–parasite interplay. Indeed, this low divergency among TcI from North and Central American region was also found when analyzing a set of *T. cruzi* isolates from the American continent by multi-locus microsatellite typing [29], supporting the results described herein and reinforcing the hypothesis that TcI originated from South America [29].

As a preliminary study assessing *T. cruzi* diversity in Panama, this study has a number of limitations. First, there is a clear geographical bias in the samples analyzed. Most of them were collected in the central region of the country (Figure 1), mostly because this area is more accessible and closer to research centers in Panama City with the technical capacity to isolate the parasite and conduct genotyping analysis. As a neglected infection throughout Latin America, many rural areas outside the central region also present suitable ecological conditions for *T. cruzi* transmission and have not been properly investigated, as demonstrated in the few isolated studies conducted in eastern and western regions of Panama [12,25,67,68]. Thus, genotyping *T. cruzi* samples from additional biological samples and wider geographic areas in the country would have allowed a better understanding of the genetic structure of *T. cruzi* in the country and exploring of the possible associations with the biology of the parasite and/or different epidemiological/pathogenic parameters. Second, it is possible that during parasite isolation and in vitro maintenance of parasites before the genotyping analysis, we missed some genetic diversity due to the clonal selection of particular lineages or intra-lineages that have overgrown the original population. In addition, DTUs isolated from blood do not necessarily reveal all DTUs in a particular host. Nevertheless, we detected different DTUs in the Panamanian samples as well as a considerable diversity within TcI isolates. Finally, there is a limited number of *T. cruzi* mitochondrial sequences from Central American countries available in public genetic databases to conduct a more robust phylogeographic regional analysis, including exploring possible association between TcI haplotypes and different host/vector species or transmission cycles.

Although our results revealed some degree of genetic heterogeneity among *T. cruzi* populations circulating in Panama, we may be far from knowing the extent of *T. cruzi* diversity in this country. This can partially be explained by the fact that simultaneous isolation of more than one population of *T. cruzi* from a single host (during potential mixed infections) is generally difficult, since different populations can produce different levels of parasitemia, growth in culture media and tissue tropism.

Additional studies that do not require isolation and culture of the parasites could help to resolve this problem. In this context, recent studies have proven that the use of complete maxicircle sequences is a superior phylogenetic marker for trypanosome taxonomic purposes and for studying the evolutionary history of trypanosomatids, resulting in phylogenetic trees with very high bootstrap support values [69,70].

## 5. Conclusions

Our multi-locus sequence typing approach using six maxicircle genes has validated the predominance of TcI in Panama across different hosts and vector species. It has also confirmed the presence of TcIII and TcVI circulating in the country. The haplotype network analysis inferred considerably genetic diversity within Panamanian TcI haplotypes and revealed a geographical structuring among TcI isolates from different endemic regions in the Americas. This study adds novelty on the genetic diversity of *T. cruzi* circulating in Panama and complements regional phylogeographic studies regarding intra-TcI variations.

## Figures and Tables

**Table 1:** List of Panamanian *Trypanosoma cruzi* isolates analyzed in this study with details about their epidemiological data, clinical history and results of discrete typing units (DTUs).

Original Code	Host/Vector (Sex, Age)	Locality (Region)	Date	Clinical Status	Outcome	*T. cruzi* DTU
TCH1	Human (Female, 9y)	Chorrera (WP)	Oct, 1980	Acute	Chronic	TcVI
TCH2	Human (Male, 78y)	Aguas Claras (Colón)	April, 1983	Asymptomatic	Indetermined	TcI
TCH3	Human (Female, 7y)	Chorrera (WP)	March, 1981	Asymptomatic	Indetermined	TcI
TCH4	Human (Male, 29y)	Chilibre (Colón)	July, l97l	Acute	Unknown	TcI
TCH5	Human (Male, 20y)	Chepo (EP)	July, 1965	Asymptomatic	Chronic	TcI
TCH6	Human (Male, 42y)	Capira (WP)	Jan, 1987	Acute	Indetermined	TcI
TCH7	Human (Female, 17y)	Mendoza (WP)	July, 1970	Asymptomatic	Indetermined	TcI
TCH8	Human (Male, 18y)	Cerro Cama (WP)	April, 1979	Asymptomatic	Unknown	TcI
TCH10	Human (Female, 13y)	Pacora (EP)	Jan, 1995	Acute	Indetermined	TcIII
TCH12	Human (Male, 31y)	Bunrunga (WP)	Oct, 2002	Acute	Unknown	TcI
TCH14	Human (Female, 1y)	Nuevo Arraiján (WP)	Oct, 1973	Acute	Chronic	TcI
TCH15	Human (Male, 3y)	Capira (WP)	Aug, 1980	Acute	Chronic	TcI
TCR1	Monkey (*Saguinus geoffroyi*)	Pacora (EP)	June, 1966	Asymptomatic	Unknown	TcI
TCR2	Opossum (*Didelphis marsupialis)*	Chepo (EP)	June, 1966	Asymptomatic	Unknown	TcI
TCR3	Otter (*Lontra longicaudis*)	Colon	May, 1973	Asymptomatic	Unknown	TcI
TCR4	Canine (*Canis familiaris*)	Chorrera (WP)	2004	Acute	Fatal	TcI
TCV1	*Panstrongylus geniculatus*	Barro Colorado (WP)	1992			TcI
TCV2	*Rhodnius pallescens*	Chilibre (Colón)	1991			TcI
TCV3	*Triatoma dimidiata*	Barro Colorado (WP)	1992			TcI
TCV4	*Rhodnius pallescens*	Cerro Cama (WP)	1985			TcI
TCV5	*Rhodnius pallescens*	Chilibre (Colón)	2011			TcI
TCV6	*Rhodnius pallescens*	Chorrera (WP)	2011			TcI
TCV7	*Rhodnius pallescens*	Chorrera (WP)	2011			TcI
TCV8	*Rhodnius pallescens*	Burunga (WP)	2011			TcI
TCV9	*Rhodnius pallescens*	Chorrera (WP)	2011			TcI
TCV10	*Rhodnius pallescens*	Chorrera (WP)	2012			TcI
TCV11	*Rhodnius pallescens*	Capira (WP)	2012			TcI
TCV12	*Rhodnius pallescens*	Santa Fe (Veraguas)	2014			TcI
TCV13	*Rhodnius pallescens*	Pedasi (Los Santos)	2016			TcI
TCV14	*Rhodnius pallescens*	Capira (WP)	2016			TcI
TCV15	*Panstrongylus geniculatus*	Montijo (Veraguas)	2015			TcIII
TCV17	*Rhodnius pallescens*	Burunga (WP)	2000			TcI

WP: Western Panama; EP: Eastern Panama; DTU: Discrete typing units.

## Data Availability

Complete sequence alignment of the concatenated genes from this study is available upon request. Accession numbers of nucleotide sequences for all gene fragments generated during this study are provided in Table S1.

## Supplementary Materials

The following supporting information can be downloaded at: https://www.mdpi.com/article/10.3390/microorganisms10020287/s1, Table S1: Accession numbers of *T. cruzi* maxicircle gene fragments reported in this study; Table S2: Haplotype regional groups (clusters) identified after analyzing concatenated mitochondrial sequences of five loci (ND1, COII, MURF1, CYT b, 12S rRNA and 9S rRNA); Table S3: Estimates of evolutionary divergency over sequence pairs between groups of haplotypes observed in this study; Table S4: Estimates of evolutionary divergence between mitochondrial sequences assessed by *p*-distance; Table S5: Number of base differences per sequence from between concatenated mitochondrial sequences.
