# Supplementary material for: Genetic Diversity of Trypanosoma cruzi in Panama Inferred by Multi-locus Sequence Typing of Mitochondrial Genes
Source: Microorganisms. 2022 Jan 26;10(2):287. doi: 10.3390/microorganisms10020287 (PMC8879757; doi:10.3390/microorganisms10020287)
Supplement: Supplementary file 1 [file microorganisms-10-00287-s001.zip › Table S2. Haplotype regional groups.pdf]

Table S2. Haplotype regional groups (clusters) identified after analyzing concatenated mitochondrial sequences of five loci (ND1, COII, MURF1, CYT b, 12S rRNA and 9S rRNA) from 90 *Trypanosma cruzi* TcI isolates, 29 from Panama and 39 reference *T. cruzi* strains from different endemic countries across the Americas; with details about the total number of haplotypes, geographical origin and the biological source of the isolates.

Regional groups	Haplotypes	Sequences	Country	Host/Vector
**Andean Region**	**Haplotype 1 (PALP20)**	PAL20	Argentina	*Didelphis albiventris*
		PAL21	Argentina	*Didelphis albiventris*
		PAL23	Argentina	*Triatoma infestans*
		PAL5	Argentina	*Didelphis albiventris*
		SJ22	Bolivia	*Didelphis marsupialis*
	**Haplotype 2 (PALP4)**	PAL4	Argentina	*Didelphis albiventris*
	**Haplotype 3 (COT38-1)**	COT38-1	Bolivia	*Akodon boliviensis*
		P234	Bolivia	*Homo sapiens*
		P238	Bolivia	*Homo sapiens*
		P268	Bolivia	*Homo sapiens*
	**Haplotype 4 (SJ37)**	SJ37	Bolivia	*Didelphis marsupialis*
		SJ12	Bolivia	*Didelphis marsupialis*
	**Haplotype 5 (SJ34)**	SJ34	Bolivia	*Didelphis marsupialis*
		SJ39	Bolivia	*Didelphis marsupialis*
		SJ41	Bolivia	*Philander opossum*
**Brazil A**	**Haplotype 6 (G-strain)**	G-strain	Brazil	Opossum
**Brazil B**	**Haplotype 7 (IM48)**	IM48	Brazil	*Didelphis marsupialis*
**Brazil C**	**Haplotype 8 (XE51)**	XE51	Brazil	*Didelphis marsupialis*
	**Haplotype 9 (XE29)**	XE29	Brazil	*Didelphis marsupialis*
	**Haplotype 10 (B20851)**	B20851	Brazil	*Didelphis marsupialis*
	**Haplotype 11 (SYLVIO X10/1)**	SILVIO	Brazil	*Homo sapiens*
**North and Central American Region and Western South American countries**	**Haplotype 12 (AAB3CL)**	AAB3CL	Colombia	*Rhodnius prolixus*
	**Haplotype 13 (SLDM1C)**	SLDM1CL8	Colombia	*Didelphis marsupialis*
		SLDM1CL8-2	Colombia	*Didelphis marsupialis*
	**Haplotype 14 (EMCL4)**	EMCL4	Colombia	*Homo sapiens*
	**Haplotype 15 (DYRCL5)**	DYRCL5	Colombia	*Homo sapiens*
	**Haplotype 16 (DAVIS)**	DAVIS	Honduras	*Triatoma dimidiata*
	**Haplotype 17 (Mexico strain)**	MEXICOSTRAIN	Mexico	Unknown
	**Haplotype 18 (TCV11)**	TCV11	Panama	*Rhodnius pallescens*
		TCR4	Panama	*Canis familiaris*
		TCH15	Panama	*Homo sapiens*
		TCH7	Panama	*Homo sapiens*
		TCV2	Panama	*Rhodnius pallescens*
		TCV7	Panama	*Rhodnius pallescens*
		TCH12	Panama	*Homo sapiens*
		TCH2	Panama	*Homo sapiens*
		TCH5	Panama	*Homo sapiens*
		TCR1	Panama	*Saguinus geoffroyi*
		TCV5	Panama	*Rhodnius pallescens*
		TCV8	Panama	*Rhodnius pallescens*

		TCV10	Panama	*Homo sapiens*
		TCH14	Panama	*Homo sapiens*
		TCH3	Panama	*Homo sapiens*
		TCH6	Panama	*Homo sapiens*
		TCR2	Panama	*Didelphis marsupialis*
		TCV6	Panama	*Rhodnius pallescens*
		TCV9	Panama	*Rhodnius pallescens*
	**Haplotype 19 (TCV13)**	TCV13	Panama	*Rhodnius pallescens*
		TCV12	Panama	*Rhodnius pallescens*
		TCV14	Panama	*Rhodnius pallescens*
	**Haplotype 20 (TCV1)**	TCV1	Panama	*Panstrongylus geniculatus*
		TCV3	Panama	*Triatoma dimidiata*
	**Haplotype 21 (TCV4)**	TCV4	Panama	*Rhodnius pallescens*
	**Haplotype 22 (TCV17)**	TCV17	Panama	*Rhodnius pallescens*
	**Haplotype 23 (TCH4)**	TCH4	Panama	*Homo sapiens*
**North and Central American Region and Western South American countries**	**Haplotype 24 (TCH8)**	TCH8	Panama	*Homo sapiens*
	**Haplotype 25 (TCR3)**	TCR3	Panama	*Lontra longicaudis*
	**Haplotype 26 (ARMA1)**	ARMA1	USA	*Dasypus novemcinctus*
		OPOS	USA	*Didelphis marsupialis*
	**Haplotype 27 (9209)**	9209	USA	*Didelphis marsupialis*
	**Haplotype 28 (KP1368)**	KP1368	El Salvador	*Homo sapiens*
	**Haplotype 29 (M13)**	M13	Venezuela	*Didelphis marsupialis*
		M16	Venezuela	*Didelphis marsupialis*
		M7	Venezuela	*Didelphis marsupialis*
	**Haplotype 30 (M18)**	M18	Venezuela	*Didelphis marsupialis*
	**Haplotype 31 (11713)**	11713	Venezuela	*Homo sapiens*
		11801	Venezuela	*Homo sapiens*
	**Haplotype 32 (1154)**	1154	Venezuela	*Homo sapiens*
	**Haplotype 33 (JR)**	JR	Venezuela	*Homo sapiens*
