# Supplementary material for: Genetic Diversity of Trypanosoma cruzi in Panama Inferred by Multi-locus Sequence Typing of Mitochondrial Genes
Source: Microorganisms. 2022 Jan 26;10(2):287. doi: 10.3390/microorganisms10020287 (PMC8879757; doi:10.3390/microorganisms10020287)
Supplement: Supplementary file 1 [file microorganisms-10-00287-s001.zip › Table S3. Estimates of Evolutionary Divergency.pdf]

Table S3. Estimates of Evolutionary Divergency over Sequence Pairs between Groups of haplotypes observed in this study: Andean Region, Brazil A, Brazil B, Brazil C and North and Central American Region & Western South American countries (NCWS). The number of base differences per sequence from averaging over all sequence pairs between groups are shown. The analysis involved 69 nucleotide sequences. Codon positions included were 1st+2nd+3rd+Noncoding. All ambiguous positions were removed for each sequence pair. There were a total of 2284 positions in the final dataset. Evolutionary analyses were conducted in MEGA X.

A. P-distance between groups of mitochondrial concatenated sequences

Brazil-A				
Brazil-B	0.015			
Brazil-C	0.023	0.015		
Andean Region	0.016	0.005	0.014	
NCWS	0.020	0.010	0.011	0.009

B. Number of nucleotide pair differences between groups of mitochondrial sequences

Brazil-A				
Brazil-B	35.0			
Brazil-C	51.8	34.0		
Andean Region	35.7	11.3	31.7	
NCWS	44.7	23.8	25.9	19.7
