# Supplementary material for: Genetic Diversity of Trypanosoma cruzi in Panama Inferred by Multi-locus Sequence Typing of Mitochondrial Genes
Source: Microorganisms. 2022 Jan 26;10(2):287. doi: 10.3390/microorganisms10020287 (PMC8879757; doi:10.3390/microorganisms10020287)
Supplement: Supplementary file 1 [file microorganisms-10-00287-s001.zip › Table S4. Divergence p distance.pdf]

Table S4. Estimates of Evolutionary Divergence between mitochondrial Sequences assessed by p-distance

1154
11713	0.000
1180-1	0.000	0.000
9209	0.002	0.001	0.001
AAB3cl3	0.005	0.005	0.005	0.005
ARMA-1	0.002	0.002	0.002	0.002	0.005
B2085-1	0.011	0.011	0.011	0.011	0.014	0.011
COT38-1	0.010	0.009	0.009	0.010	0.012	0.009	0.013
DAVIS	0.001	0.000	0.000	0.001	0.004	0.001	0.010	0.009
DYRcl5	0.001	0.001	0.001	0.002	0.005	0.003	0.012	0.010	0.001
EMcl4	0.001	0.000	0.000	0.002	0.004	0.002	0.011	0.010	0.001	0.000
G-strain	0.020	0.020	0.020	0.020	0.023	0.019	0.022	0.018	0.019	0.021	0.020
IM48	0.011	0.011	0.011	0.011	0.013	0.011	0.015	0.008	0.010	0.011	0.011	0.015
JR	0.002	0.002	0.002	0.002	0.005	0.002	0.011	0.009	0.001	0.003	0.002	0.020	0.011
KP136828	0.001	0.000	0.000	0.001	0.004	0.001	0.011	0.009	0.000	0.001	0.001	0.019	0.010	0.001
M13	0.002	0.002	0.002	0.002	0.003	0.002	0.011	0.009	0.001	0.003	0.002	0.020	0.011	0.002	0.001
M16	0.002	0.002	0.002	0.002	0.003	0.002	0.011	0.009	0.001	0.003	0.002	0.020	0.011	0.002	0.001	0.000
M18	0.003	0.002	0.002	0.003	0.004	0.002	0.011	0.010	0.002	0.003	0.003	0.020	0.011	0.002	0.002	0.000	0.000
M7	0.002	0.002	0.002	0.002	0.003	0.002	0.011	0.009	0.001	0.003	0.002	0.020	0.011	0.002	0.001	0.000	0.000	0.000
Mexico-strain	0.001	0.000	0.000	0.001	0.004	0.001	0.011	0.009	0.000	0.001	0.001	0.019	0.010	0.001	0.000	0.001	0.001	0.002	0.001
OPOS	0.002	0.002	0.002	0.002	0.005	0.000	0.011	0.009	0.001	0.003	0.002	0.019	0.011	0.002	0.001	0.002	0.002	0.002	0.002	0.001
P234	0.010	0.009	0.009	0.010	0.012	0.009	0.013	0.000	0.009	0.010	0.010	0.018	0.008	0.009	0.009	0.009	0.009	0.010	0.009	0.009	0.009
P238	0.010	0.009	0.009	0.010	0.012	0.009	0.013	0.000	0.009	0.010	0.010	0.018	0.008	0.009	0.009	0.009	0.009	0.010	0.009	0.009	0.009	0.000
P268	0.010	0.009	0.009	0.010	0.012	0.009	0.013	0.000	0.009	0.010	0.010	0.018	0.008	0.009	0.009	0.009	0.009	0.010	0.009	0.009	0.009	0.000	0.000
PAL20	0.009	0.009	0.009	0.009	0.011	0.009	0.014	0.007	0.008	0.010	0.009	0.014	0.004	0.009	0.008	0.009	0.009	0.009	0.009	0.008	0.009	0.007	0.007	0.007
PAL21	0.009	0.009	0.009	0.009	0.011	0.009	0.014	0.007	0.008	0.010	0.009	0.014	0.004	0.009	0.008	0.009	0.009	0.009	0.009	0.008	0.009	0.007	0.007	0.007	0.000
PAL23	0.009	0.009	0.009	0.009	0.011	0.009	0.014	0.007	0.008	0.010	0.009	0.014	0.004	0.009	0.008	0.009	0.009	0.009	0.009	0.008	0.009	0.007	0.007	0.007	0.000	0.000
PAL4	0.007	0.007	0.007	0.007	0.009	0.007	0.013	0.009	0.006	0.007	0.007	0.017	0.006	0.007	0.006	0.007	0.007	0.007	0.007	0.006	0.007	0.009	0.009	0.009	0.002	0.002	0.002
PAL5	0.009	0.009	0.009	0.009	0.011	0.009	0.014	0.007	0.008	0.010	0.009	0.014	0.004	0.009	0.008	0.009	0.009	0.009	0.009	0.008	0.009	0.007	0.007	0.007	0.000	0.000	0.000	0.002
Silvio	0.013	0.013	0.013	0.014	0.016	0.013	0.004	0.016	0.013	0.014	0.014	0.025	0.017	0.013	0.013	0.013	0.013	0.014	0.013	0.013	0.013	0.016	0.016	0.016	0.016	0.016	0.016	0.016	0.016
SJ12	0.009	0.009	0.009	0.009	0.011	0.009	0.014	0.007	0.008	0.010	0.009	0.014	0.004	0.009	0.008	0.009	0.009	0.009	0.009	0.008	0.009	0.007	0.007	0.007	0.001	0.001	0.001	0.003	0.001	0.016
SJ22	0.009	0.009	0.009	0.009	0.011	0.009	0.014	0.007	0.008	0.010	0.009	0.014	0.004	0.009	0.008	0.009	0.009	0.009	0.009	0.008	0.009	0.007	0.007	0.007	0.000	0.000	0.000	0.002	0.000	0.016	0.001
SJ34	0.009	0.009	0.009	0.009	0.011	0.009	0.014	0.007	0.008	0.010	0.009	0.014	0.004	0.009	0.008	0.009	0.009	0.009	0.009	0.008	0.009	0.007	0.007	0.007	0.000	0.000	0.000	0.002	0.000	0.016	0.001	0.000
SJ37	0.009	0.009	0.009	0.009	0.011	0.009	0.014	0.007	0.008	0.010	0.009	0.014	0.004	0.009	0.008	0.009	0.009	0.009	0.009	0.008	0.009	0.007	0.007	0.007	0.001	0.001	0.001	0.003	0.001	0.016	0.000	0.001	0.001
SJ39	0.009	0.009	0.009	0.009	0.011	0.009	0.014	0.007	0.008	0.010	0.009	0.014	0.004	0.009	0.008	0.009	0.009	0.009	0.009	0.008	0.009	0.007	0.007	0.007	0.000	0.000	0.000	0.002	0.000	0.016	0.001	0.000	0.000	0.001
SJ41	0.009	0.009	0.009	0.009	0.011	0.009	0.014	0.007	0.008	0.010	0.009	0.014	0.004	0.009	0.008	0.009	0.009	0.009	0.009	0.008	0.009	0.007	0.007	0.007	0.000	0.000	0.000	0.002	0.000	0.016	0.001	0.000	0.000	0.001	0.000
SLDm1cl8	0.003	0.002	0.002	0.003	0.004	0.002	0.011	0.010	0.002	0.002	0.002	0.020	0.011	0.002	0.002	0.001	0.001	0.002	0.001	0.002	0.002	0.010	0.010	0.010	0.009	0.009	0.009	0.007	0.009	0.014	0.009	0.009	0.009	0.009	0.009	0.009
SLDm1cl8(2)	0.003	0.002	0.002	0.003	0.004	0.002	0.011	0.010	0.002	0.002	0.002	0.020	0.011	0.002	0.002	0.001	0.001	0.002	0.001	0.002	0.002	0.010	0.010	0.010	0.009	0.009	0.009	0.007	0.009	0.014	0.009	0.009	0.009	0.009	0.009	0.009	0.000
TCH12	0.002	0.001	0.001	0.002	0.004	0.001	0.011	0.009	0.001	0.002	0.002	0.019	0.010	0.001	0.001	0.001	0.001	0.002	0.001	0.001	0.001	0.009	0.009	0.009	0.008	0.008	0.008	0.006	0.008	0.013	0.008	0.008	0.008	0.008	0.008	0.008	0.002	0.002
TCH14	0.002	0.001	0.001	0.002	0.004	0.001	0.011	0.009	0.001	0.002	0.002	0.019	0.010	0.001	0.001	0.001	0.001	0.002	0.001	0.001	0.001	0.009	0.009	0.009	0.008	0.008	0.008	0.006	0.008	0.013	0.008	0.008	0.008	0.008	0.008	0.008	0.002	0.002	0.000
TCH15	0.002	0.001	0.001	0.002	0.004	0.001	0.011	0.009	0.001	0.002	0.002	0.019	0.010	0.001	0.001	0.001	0.001	0.002	0.001	0.001	0.001	0.009	0.009	0.009	0.008	0.008	0.008	0.006	0.008	0.013	0.008	0.008	0.008	0.008	0.008	0.008	0.002	0.002	0.000	0.000
TCH2	0.002	0.001	0.001	0.002	0.004	0.001	0.011	0.009	0.001	0.002	0.002	0.019	0.010	0.001	0.001	0.001	0.001	0.002	0.001	0.001	0.001	0.009	0.009	0.009	0.008	0.008	0.008	0.006	0.008	0.013	0.008	0.008	0.008	0.008	0.008	0.008	0.002	0.002	0.000	0.000	0.000
TCH3	0.002	0.001	0.001	0.002	0.004	0.001	0.011	0.009	0.001	0.002	0.002	0.019	0.010	0.001	0.001	0.001	0.001	0.002	0.001	0.001	0.001	0.009	0.009	0.009	0.008	0.008	0.008	0.006	0.008	0.013	0.008	0.008	0.008	0.008	0.008	0.008	0.002	0.002	0.000	0.000	0.000	0.000
TCH4	0.002	0.001	0.001	0.002	0.004	0.001	0.011	0.009	0.001	0.002	0.002	0.019	0.010	0.001	0.001	0.001	0.001	0.002	0.001	0.001	0.001	0.009	0.009	0.009	0.008	0.008	0.008	0.006	0.008	0.013	0.008	0.008	0.008	0.008	0.008	0.008	0.002	0.002	0.000	0.000	0.000	0.000	0.000
TCH5	0.002	0.001	0.001	0.002	0.004	0.001	0.011	0.009	0.001	0.002	0.002	0.019	0.010	0.001	0.001	0.001	0.001	0.002	0.001	0.001	0.001	0.009	0.009	0.009	0.008	0.008	0.008	0.006	0.008	0.013	0.008	0.008	0.008	0.008	0.008	0.008	0.002	0.002	0.000	0.000	0.000	0.000	0.000	0.000
TCH6	0.002	0.001	0.001	0.002	0.004	0.001	0.011	0.009	0.001	0.002	0.002	0.019	0.010	0.001	0.001	0.001	0.001	0.002	0.001	0.001	0.001	0.009	0.009	0.009	0.008	0.008	0.008	0.006	0.008	0.013	0.008	0.008	0.008	0.008	0.008	0.008	0.002	0.002	0.000	0.000	0.000	0.000	0.000	0.000	0.000
TCH7	0.002	0.001	0.001	0.002	0.004	0.001	0.011	0.009	0.001	0.002	0.002	0.019	0.010	0.001	0.001	0.001	0.001	0.002	0.001	0.001	0.001	0.009	0.009	0.009	0.008	0.008	0.008	0.006	0.008	0.013	0.008	0.008	0.008	0.008	0.008	0.008	0.002	0.002	0.000	0.000	0.000	0.000	0.000	0.000	0.000	0.000
TCH8	0.002	0.002	0.002	0.002	0.005	0.002	0.011	0.009	0.001	0.003	0.002	0.020	0.011	0.002	0.001	0.002	0.002	0.002	0.002	0.001	0.002	0.009	0.009	0.009	0.009	0.009	0.009	0.007	0.009	0.013	0.009	0.009	0.009	0.009	0.009	0.009	0.002	0.002	0.000	0.000	0.000	0.000	0.000	0.000	0.000	0.000	0.000
TCR1	0.002	0.001	0.001	0.002	0.004	0.001	0.011	0.009	0.001	0.002	0.002	0.019	0.010	0.001	0.001	0.001	0.001	0.002	0.001	0.001	0.001	0.009	0.009	0.009	0.008	0.008	0.008	0.006	0.008	0.013	0.008	0.008	0.008	0.008	0.008	0.008	0.002	0.002	0.000	0.000	0.000	0.000	0.000	0.000	0.000	0.000	0.000	0.000
TCR2	0.002	0.001	0.001	0.002	0.004	0.001	0.011	0.009	0.001	0.002	0.002	0.019	0.010	0.001	0.001	0.001	0.001	0.002	0.001	0.001	0.001	0.009	0.009	0.009	0.008	0.008	0.008	0.006	0.008	0.013	0.008	0.008	0.008	0.008	0.008	0.008	0.002	0.002	0.000	0.000	0.000	0.000	0.000	0.000	0.000	0.000	0.000	0.000	0.000
TCR3	0.002	0.001	0.001	0.002	0.004	0.001	0.011	0.009	0.001	0.002	0.002	0.019	0.010	0.001	0.001	0.001	0.001	0.002	0.001	0.001	0.001	0.009	0.009	0.009	0.008	0.008	0.008	0.006	0.008	0.013	0.008	0.008	0.008	0.008	0.008	0.008	0.002	0.002	0.001	0.001	0.001	0.001	0.001	0.001	0.001	0.001	0.001	0.001	0.001	0.001
TCR4	0.002	0.001	0.001	0.002	0.004	0.001	0.011	0.009	0.001	0.002	0.002	0.019	0.010	0.001	0.001	0.001	0.001	0.002	0.001	0.001	0.001	0.009	0.009	0.009	0.008	0.008	0.008	0.006	0.008	0.013	0.008	0.008	0.008	0.008	0.008	0.008	0.002	0.002	0.000	0.000	0.000	0.000	0.000	0.000	0.000	0.000	0.000	0.000	0.000	0.000	0.001
TCV1	0.002	0.002	0.002	0.002	0.005	0.002	0.011	0.009	0.001	0.003	0.002	0.020	0.011	0.002	0.001	0.002	0.002	0.002	0.002	0.001	0.002	0.009	0.009	0.009	0.008	0.008	0.008	0.006	0.008	0.013	0.008	0.008	0.008	0.008	0.008	0.008	0.002	0.002	0.000	0.000	0.000	0.000	0.000	0.000	0.000	0.000	0.000	0.001	0.000	0.000	0.001	0.000
TCV10	0.002	0.001	0.001	0.002	0.004	0.001	0.011	0.009	0.001	0.002	0.002	0.019	0.010	0.001	0.001	0.001	0.001	0.002	0.001	0.001	0.001	0.009	0.009	0.009	0.008	0.008	0.008	0.006	0.008	0.013	0.008	0.008	0.008	0.008	0.008	0.008	0.002	0.002	0.000	0.000	0.000	0.000	0.000	0.000	0.000	0.000	0.000	0.000	0.000	0.000	0.001	0.000	0.000
TCV11	0.002	0.001	0.001	0.002	0.004	0.001	0.011	0.009	0.001	0.002	0.002	0.019	0.010	0.001	0.001	0.001	0.001	0.002	0.001	0.001	0.001	0.009	0.009	0.009	0.008	0.008	0.008	0.006	0.008	0.013	0.008	0.008	0.008	0.008	0.008	0.008	0.002	0.002	0.000	0.000	0.000	0.000	0.000	0.000	0.000	0.000	0.000	0.000	0.000	0.000	0.001	0.000	0.000	0.000
TCV12	0.002	0.001	0.001	0.002	0.004	0.001	0.011	0.009	0.001	0.002	0.002	0.019	0.010	0.001	0.001	0.001	0.001	0.002	0.001	0.001	0.001	0.009	0.009	0.009	0.008	0.008	0.008	0.006	0.008	0.013	0.008	0.008	0.008	0.008	0.008	0.008	0.002	0.002	0.000	0.000	0.000	0.000	0.000	0.000	0.000	0.000	0.000	0.000	0.000	0.000	0.001	0.000	0.000	0.000	0.000
TCV13	0.002	0.001	0.001	0.002	0.004	0.001	0.011	0.009	0.001	0.002	0.002	0.019	0.010	0.001	0.001	0.001	0.001	0.002	0.001	0.001	0.001	0.009	0.009	0.009	0.008	0.008	0.008	0.006	0.008	0.013	0.008	0.008	0.008	0.008	0.008	0.008	0.002	0.002	0.000	0.000	0.000	0.000	0.000	0.000	0.000	0.000	0.000	0.000	0.000	0.000	0.001	0.000	0.000	0.000	0.000	0.000
TCV14	0.002	0.001	0.001	0.002	0.004	0.001	0.011	0.009	0.001	0.002	0.002	0.019	0.010	0.001	0.001	0.001	0.001	0.002	0.001	0.001	0.001	0.009	0.009	0.009	0.008	0.008	0.008	0.006	0.008	0.013	0.008	0.008	0.008	0.008	0.008	0.008	0.002	0.002	0.000	0.000	0.000	0.000	0.000	0.000	0.000	0.000	0.000	0.000	0.000	0.000	0.001	0.000	0.000	0.000	0.000	0.000	0.000
TCV17	0.004	0.003	0.003	0.004	0.006	0.003	0.012	0.011	0.003	0.004	0.004	0.021	0.012	0.003	0.003	0.003	0.003	0.003	0.003	0.003	0.003	0.011	0.011	0.011	0.010	0.010	0.010	0.008	0.010	0.015	0.010	0.010	0.010	0.010	0.010	0.010	0.004	0.004	0.002	0.002	0.002	0.002	0.002	0.002	0.002	0.002	0.002	0.002	0.002	0.002	0.003	0.002	0.002	0.002	0.002	0.002	0.002	0.002
TCV2	0.002	0.001	0.001	0.002	0.004	0.001	0.011	0.009	0.001	0.002	0.002	0.019	0.010	0.001	0.001	0.001	0.001	0.002	0.001	0.001	0.001	0.009	0.009	0.009	0.008	0.008	0.008	0.006	0.008	0.013	0.008	0.008	0.008	0.008	0.008	0.008	0.002	0.002	0.000	0.000	0.000	0.000	0.000	0.000	0.000	0.000	0.000	0.000	0.000	0.000	0.001	0.000	0.000	0.000	0.000	0.000	0.000	0.000	0.002
TCV3	0.002	0.002	0.002	0.002	0.005	0.002	0.011	0.009	0.001	0.003	0.002	0.020	0.011	0.002	0.001	0.002	0.002	0.002	0.002	0.001	0.002	0.009	0.009	0.009	0.008	0.008	0.008	0.006	0.008	0.013	0.008	0.008	0.008	0.008	0.008	0.008	0.002	0.002	0.000	0.000	0.000	0.000	0.000	0.000	0.000	0.000	0.000	0.001	0.000	0.000	0.001	0.000	0.000	0.000	0.000	0.000	0.000	0.000	0.002	0.000
TCV4	0.002	0.002	0.002	0.002	0.005	0.002	0.011	0.009	0.001	0.003	0.002	0.020	0.011	0.002	0.001	0.002	0.002	0.002	0.002	0.001	0.002	0.009	0.009	0.009	0.009	0.009	0.009	0.007	0.009	0.013	0.009	0.009	0.009	0.009	0.009	0.009	0.002	0.002	0.000	0.000	0.000	0.000	0.000	0.000	0.000	0.000	0.000	0.001	0.000	0.000	0.001	0.000	0.001	0.000	0.000	0.000	0.000	0.000	0.002	0.000	0.001
TCV5	0.002	0.001	0.001	0.002	0.004	0.001	0.011	0.009	0.001	0.002	0.002	0.019	0.010	0.001	0.001	0.001	0.001	0.002	0.001	0.001	0.001	0.009	0.009	0.009	0.008	0.008	0.008	0.006	0.008	0.013	0.008	0.008	0.008	0.008	0.008	0.008	0.002	0.002	0.000	0.000	0.000	0.000	0.000	0.000	0.000	0.000	0.000	0.000	0.000	0.000	0.001	0.000	0.000	0.000	0.000	0.000	0.000	0.000	0.002	0.000	0.000	0.000
TCV6	0.002	0.001	0.001	0.002	0.004	0.001	0.011	0.009	0.001	0.002	0.002	0.019	0.010	0.001	0.001	0.001	0.001	0.002	0.001	0.001	0.001	0.009	0.009	0.009	0.008	0.008	0.008	0.006	0.008	0.013	0.008	0.008	0.008	0.008	0.008	0.008	0.002	0.002	0.000	0.000	0.000	0.000	0.000	0.000	0.000	0.000	0.000	0.000	0.000	0.000	0.001	0.000	0.000	0.000	0.000	0.000	0.000	0.000	0.002	0.000	0.000	0.000	0.000
TCV7	0.002	0.001	0.001	0.002	0.004	0.001	0.011	0.009	0.001	0.002	0.002	0.019	0.010	0.001	0.001	0.001	0.001	0.002	0.001	0.001	0.001	0.009	0.009	0.009	0.008	0.008	0.008	0.006	0.008	0.013	0.008	0.008	0.008	0.008	0.008	0.008	0.002	0.002	0.000	0.000	0.000	0.000	0.000	0.000	0.000	0.000	0.000	0.000	0.000	0.000	0.001	0.000	0.000	0.000	0.000	0.000	0.000	0.000	0.002	0.000	0.000	0.000	0.000	0.000
TCV8	0.002	0.001	0.001	0.002	0.004	0.001	0.011	0.009	0.001	0.002	0.002	0.019	0.010	0.001	0.001	0.001	0.001	0.002	0.001	0.001	0.001	0.009	0.009	0.009	0.008	0.008	0.008	0.006	0.008	0.013	0.008	0.008	0.008	0.008	0.008	0.008	0.002	0.002	0.000	0.000	0.000	0.000	0.000	0.000	0.000	0.000	0.000	0.000	0.000	0.000	0.001	0.000	0.000	0.000	0.000	0.000	0.000	0.000	0.002	0.000	0.000	0.000	0.000	0.000	0.000
TCV9	0.002	0.001	0.001	0.002	0.004	0.001	0.011	0.009	0.001	0.002	0.002	0.019	0.010	0.001	0.001	0.001	0.001	0.002	0.001	0.001	0.001	0.009	0.009	0.009	0.008	0.008	0.008	0.006	0.008	0.013	0.008	0.008	0.008	0.008	0.008	0.008	0.002	0.002	0.000	0.000	0.000	0.000	0.000	0.000	0.000	0.000	0.000	0.000	0.000	0.000	0.001	0.000	0.000	0.000	0.000	0.000	0.000	0.000	0.002	0.000	0.000	0.000	0.000	0.000	0.000	0.000
XE29	0.011	0.011	0.011	0.012	0.015	0.011	0.003	0.013	0.011	0.013	0.012	0.022	0.015	0.012	0.011	0.011	0.011	0.012	0.011	0.011	0.011	0.013	0.013	0.013	0.014	0.014	0.014	0.014	0.014	0.006	0.014	0.014	0.014	0.014	0.014	0.014	0.012	0.012	0.011	0.011	0.011	0.011	0.011	0.011	0.011	0.011	0.011	0.012	0.011	0.011	0.011	0.011	0.012	0.011	0.011	0.011	0.011	0.011	0.013	0.011	0.012	0.012	0.011	0.011	0.011	0.011	0.011
XE51	0.010	0.010	0.010	0.010	0.013	0.010	0.001	0.012	0.009	0.011	0.011	0.022	0.014	0.010	0.010	0.010	0.010	0.010	0.010	0.010	0.010	0.012	0.012	0.012	0.013	0.013	0.013	0.012	0.013	0.004	0.013	0.013	0.013	0.013	0.013	0.013	0.011	0.011	0.010	0.010	0.010	0.010	0.010	0.010	0.010	0.010	0.010	0.010	0.010	0.010	0.010	0.010	0.010	0.010	0.010	0.010	0.010	0.010	0.011	0.010	0.010	0.010	0.010	0.010	0.010	0.010	0.010	0.002
