# Supplementary material for: Genetic Diversity of Trypanosoma cruzi in Panama Inferred by Multi-locus Sequence Typing of Mitochondrial Genes
Source: Microorganisms. 2022 Jan 26;10(2):287. doi: 10.3390/microorganisms10020287 (PMC8879757; doi:10.3390/microorganisms10020287)
Supplement: Supplementary file 1 [file microorganisms-10-00287-s001.zip › Table S5. Number of base differences per sequence.pdf]

Table S5. Number of base differences per sequence from between concatenated mitochondrial sequences.

1154																																																																				
11713	1.0																																																																		
1180-1	1.0	0.0																																																																	
9209	4.0	3.0	3.0																																																																
AAB3cl3	12.0	11.0	11.0	12.0																																																															
ARMA-1	5.0	4.0	4.0	5.0	11.0																																																													
B2085-1	24.0	24.0	24.0	25.0	32.0	24.0																																																											
COT38-1	22.0	21.0	21.0	22.0	28.0	21.0	30.0																																																										
DAVIS	2.0	1.0	1.0	2.0	10.0	3.0	23.0	20.0																																																								
DYRcl5	3.0	2.0	2.0	5.0	11.0	6.0	27.0	23.0	3.0																																																							
EMcl4	2.0	1.0	1.0	4.0	10.0	5.0	26.0	22.0	2.0	1.0																																																						
G-strain	46.0	45.0	45.0	46.0	52.0	43.0	51.0	42.0	44.0	47.0	46.0																																																				
IM48	25.0	24.0	24.0	25.0	29.0	24.0	33.0	19.0	23.0	26.0	25.0	35.0																																																			
JR	5.0	4.0	4.0	5.0	11.0	4.0	25.0	21.0	3.0	6.0	5.0	45.0	24.0																																																		
KP136828	2.0	1.0	1.0	2.0	10.0	3.0	24.0	20.0	0.0	3.0	2.0	44.0	23.0	3.0																																																	
M13	5.0	4.0	4.0	5.0	7.0	4.0	24.0	21.0	3.0	6.0	5.0	45.0	24.0	4.0	3.0																																																
M16	5.0	4.0	4.0	5.0	7.0	4.0	24.0	21.0	3.0	6.0	5.0	45.0	24.0	4.0	3.0	0.0																																															
M18	6.0	5.0	5.0	6.0	8.0	5.0	25.0	22.0	4.0	7.0	6.0	46.0	24.0	5.0	4.0	1.0	1.0																																														
M7	5.0	4.0	4.0	5.0	7.0	4.0	24.0	21.0	3.0	6.0	5.0	45.0	24.0	4.0	3.0	0.0	0.0	1.0																																													
Mexico-strain	2.0	1.0	1.0	2.0	10.0	3.0	24.0	20.0	0.0	3.0	2.0	44.0	23.0	3.0	0.0	3.0	3.0	4.0	3.0																																												
OPOS	5.0	4.0	4.0	5.0	11.0	0.0	24.0	21.0	3.0	6.0	5.0	43.0	24.0	4.0	3.0	4.0	4.0	5.0	4.0	3.0																																										
P234	22.0	21.0	21.0	22.0	28.0	21.0	30.0	0.0	20.0	23.0	22.0	42.0	19.0	21.0	20.0	21.0	21.0	22.0	21.0	20.0	21.0																																									
P238	22.0	21.0	21.0	22.0	28.0	21.0	30.0	0.0	20.0	23.0	22.0	42.0	19.0	21.0	20.0	21.0	21.0	22.0	21.0	20.0	21.0	0.0																																								
P268	22.0	21.0	21.0	22.0	28.0	21.0	30.0	0.0	20.0	23.0	22.0	42.0	19.0	21.0	20.0	21.0	21.0	22.0	21.0	20.0	21.0	0.0	0.0																																							
PAL20	21.0	20.0	20.0	21.0	25.0	20.0	31.0	17.0	19.0	22.0	21.0	33.0	8.0	20.0	19.0	20.0	20.0	21.0	20.0	19.0	20.0	17.0	17.0	17.0																																						
PAL21	21.0	20.0	20.0	21.0	25.0	20.0	31.0	17.0	19.0	22.0	21.0	33.0	8.0	20.0	19.0	20.0	20.0	21.0	20.0	19.0	20.0	17.0	17.0	17.0	0.0																																					
PAL23	21.0	20.0	20.0	21.0	25.0	20.0	31.0	17.0	19.0	22.0	21.0	33.0	8.0	20.0	19.0	20.0	20.0	21.0	20.0	19.0	20.0	17.0	17.0	17.0	0.0	0.0																																				
PAL4	16.0	15.0	15.0	16.0	20.0	15.0	30.0	20.0	14.0	17.0	16.0	38.0	13.0	15.0	14.0	15.0	15.0	16.0	15.0	14.0	15.0	20.0	20.0	20.0	5.0	5.0	5.0																																			
PAL5	21.0	20.0	20.0	21.0	25.0	20.0	31.0	17.0	19.0	22.0	21.0	33.0	8.0	20.0	19.0	20.0	20.0	21.0	20.0	19.0	20.0	17.0	17.0	17.0	0.0	0.0	0.0	5.0																																		
Silvio	30.0	30.0	30.0	31.0	37.0	30.0	8.0	36.0	29.0	32.0	31.0	56.0	39.0	30.0	29.0	30.0	30.0	31.0	30.0	29.0	30.0	36.0	36.0	36.0	37.0	37.0	37.0	36.0	37.0																																	
SJ12	21.0	20.0	20.0	21.0	25.0	20.0	31.0	17.0	19.0	22.0	21.0	33.0	8.0	20.0	19.0	20.0	20.0	21.0	20.0	19.0	20.0	17.0	17.0	17.0	2.0	2.0	2.0	7.0	2.0	37.0																																
SJ22	21.0	20.0	20.0	21.0	25.0	20.0	31.0	17.0	19.0	22.0	21.0	33.0	8.0	20.0	19.0	20.0	20.0	21.0	20.0	19.0	20.0	17.0	17.0	17.0	0.0	0.0	0.0	5.0	0.0	37.0	2.0																															
SJ34	21.0	20.0	20.0	21.0	25.0	20.0	31.0	17.0	19.0	22.0	21.0	33.0	8.0	20.0	19.0	20.0	20.0	21.0	20.0	19.0	20.0	17.0	17.0	17.0	0.0	0.0	0.0	5.0	0.0	37.0	2.0	0.0																														
SJ37	21.0	20.0	20.0	21.0	25.0	20.0	31.0	17.0	19.0	22.0	21.0	33.0	8.0	20.0	19.0	20.0	20.0	21.0	20.0	19.0	20.0	17.0	17.0	17.0	2.0	2.0	2.0	7.0	2.0	37.0	0.0	2.0	2.0																																			
SJ39	21.0	20.0	20.0	21.0	25.0	20.0	31.0	17.0	19.0	22.0	21.0	33.0	8.0	20.0	19.0	20.0	20.0	21.0	20.0	19.0	20.0	17.0	17.0	17.0	0.0	0.0	0.0	5.0	0.0	37.0	2.0	0.0	0.0	2.0																																		
SJ41	21.0	20.0	20.0	21.0	25.0	20.0	31.0	17.0	19.0	22.0	21.0	33.0	8.0	20.0	19.0	20.0	20.0	21.0	20.0	19.0	20.0	17.0	17.0	17.0	0.0	0.0	0.0	5.0	0.0	37.0	2.0	0.0	0.0	2.0	0.0																																	
SLDm1cl8	6.0	5.0	5.0	6.0	8.0	5.0	26.0	22.0	4.0	5.0	4.0	46.0	25.0	5.0	4.0	3.0	3.0	4.0	3.0	4.0	5.0	22.0	22.0	22.0	21.0	21.0	21.0	16.0	21.0	31.0	21.0	21.0	21.0	21.0	21.0	21.0																																
SLDm1cl8(2)	6.0	5.0	5.0	6.0	8.0	5.0	26.0	22.0	4.0	5.0	4.0	46.0	25.0	5.0	4.0	3.0	3.0	4.0	3.0	4.0	5.0	22.0	22.0	22.0	21.0	21.0	21.0	16.0	21.0	31.0	21.0	21.0	21.0	21.0	21.0	21.0	0.0																															
TCH12	4.0	3.0	3.0	4.0	10.0	3.0	24.0	20.0	2.0	5.0	4.0	44.0	23.0	3.0	2.0	3.0	3.0	4.0	3.0	2.0	3.0	20.0	20.0	20.0	19.0	19.0	19.0	14.0	19.0	29.0	19.0	19.0	19.0	19.0	19.0	19.0	4.0	4.0																														
TCH14	4.0	3.0	3.0	4.0	10.0	3.0	24.0	20.0	2.0	5.0	4.0	44.0	23.0	3.0	2.0	3.0	3.0	4.0	3.0	2.0	3.0	20.0	20.0	20.0	19.0	19.0	19.0	14.0	19.0	29.0	19.0	19.0	19.0	19.0	19.0	19.0	4.0	4.0	0.0																													
TCH15	4.0	3.0	3.0	4.0	10.0	3.0	24.0	20.0	2.0	5.0	4.0	44.0	23.0	3.0	2.0	3.0	3.0	4.0	3.0	2.0	3.0	20.0	20.0	20.0	19.0	19.0	19.0	14.0	19.0	29.0	19.0	19.0	19.0	19.0	19.0	19.0	4.0	4.0	0.0	0.0																												
TCH2	4.0	3.0	3.0	4.0	10.0	3.0	24.0	20.0	2.0	5.0	4.0	44.0	23.0	3.0	2.0	3.0	3.0	4.0	3.0	2.0	3.0	20.0	20.0	20.0	19.0	19.0	19.0	14.0	19.0	29.0	19.0	19.0	19.0	19.0	19.0	19.0	4.0	4.0	0.0	0.0	0.0																											
TCH3	4.0	3.0	3.0	4.0	10.0	3.0	24.0	20.0	2.0	5.0	4.0	44.0	23.0	3.0	2.0	3.0	3.0	4.0	3.0	2.0	3.0	20.0	20.0	20.0	19.0	19.0	19.0	14.0	19.0	29.0	19.0	19.0	19.0	19.0	19.0	19.0	4.0	4.0	0.0	0.0	0.0	0.0																										
TCH4	4.0	3.0	3.0	4.0	10.0	3.0	24.0	20.0	2.0	5.0	4.0	44.0	23.0	3.0	2.0	3.0	3.0	4.0	3.0	2.0	3.0	20.0	20.0	20.0	19.0	19.0	19.0	14.0	19.0	29.0	19.0	19.0	19.0	19.0	19.0	19.0	4.0	4.0	0.0	0.0	0.0	0.0	0.0																									
TCH5	4.0	3.0	3.0	4.0	10.0	3.0	24.0	20.0	2.0	5.0	4.0	44.0	23.0	3.0	2.0	3.0	3.0	4.0	3.0	2.0	3.0	20.0	20.0	20.0	19.0	19.0	19.0	14.0	19.0	29.0	19.0	19.0	19.0	19.0	19.0	19.0	4.0	4.0	0.0	0.0	0.0	0.0	0.0	0.0																								
TCH6	4.0	3.0	3.0	4.0	10.0	3.0	24.0	20.0	2.0	5.0	4.0	44.0	23.0	3.0	2.0	3.0	3.0	4.0	3.0	2.0	3.0	20.0	20.0	20.0	19.0	19.0	19.0	14.0	19.0	29.0	19.0	19.0	19.0	19.0	19.0	19.0	4.0	4.0	0.0	0.0	0.0	0.0	0.0	0.0	0.0																							
TCH7	4.0	3.0	3.0	4.0	10.0	3.0	24.0	20.0	2.0	5.0	4.0	44.0	23.0	3.0	2.0	3.0	3.0	4.0	3.0	2.0	3.0	20.0	20.0	20.0	19.0	19.0	19.0	14.0	19.0	29.0	19.0	19.0	19.0	19.0	19.0	19.0	4.0	4.0	0.0	0.0	0.0	0.0	0.0	0.0	0.0	0.0																						
TCH8	5.0	4.0	4.0	5.0	11.0	4.0	25.0	21.0	3.0	6.0	5.0	45.0	24.0	4.0	3.0	4.0	4.0	5.0	4.0	3.0	4.0	21.0	21.0	21.0	20.0	20.0	20.0	15.0	20.0	30.0	20.0	20.0	20.0	20.0	20.0	20.0	5.0	5.0	1.0	1.0	1.0	1.0	1.0	1.0	1.0	1.0	1.0																					
TCR1	4.0	3.0	3.0	4.0	10.0	3.0	24.0	20.0	2.0	5.0	4.0	44.0	23.0	3.0	2.0	3.0	3.0	4.0	3.0	2.0	3.0	20.0	20.0	20.0	19.0	19.0	19.0	14.0	19.0	29.0	19.0	19.0	19.0	19.0	19.0	19.0	4.0	4.0	0.0	0.0	0.0	0.0	0.0	0.0	0.0	0.0	0.0	1.0																				
TCR2	4.0	3.0	3.0	4.0	10.0	3.0	24.0	20.0	2.0	5.0	4.0	44.0	23.0	3.0	2.0	3.0	3.0	4.0	3.0	2.0	3.0	20.0	20.0	20.0	19.0	19.0	19.0	14.0	19.0	29.0	19.0	19.0	19.0	19.0	19.0	19.0	4.0	4.0	0.0	0.0	0.0	0.0	0.0	0.0	0.0	0.0	0.0	1.0	0.0																			
TCR3	4.0	3.0	3.0	4.0	10.0	3.0	24.0	20.0	2.0	5.0	4.0	44.0	23.0	3.0	2.0	3.0	3.0	4.0	3.0	2.0	3.0	20.0	20.0	20.0	19.0	19.0	19.0	14.0	19.0	29.0	19.0	19.0	19.0	19.0	19.0	19.0	4.0	4.0	2.0	2.0	2.0	2.0	2.0	2.0	2.0	2.0	2.0	3.0	2.0	2.0																		
TCR4	4.0	3.0	3.0	4.0	10.0	3.0	24.0	20.0	2.0	5.0	4.0	44.0	23.0	3.0	2.0	3.0	3.0	4.0	3.0	2.0	3.0	20.0	20.0	20.0	19.0	19.0	19.0	14.0	19.0	29.0	19.0	19.0	19.0	19.0	19.0	19.0	4.0	4.0	0.0	0.0	0.0	0.0	0.0	0.0	0.0	0.0	0.0	1.0	0.0	0.0	2.0																	
TCV1	5.0	4.0	4.0	5.0	11.0	4.0	25.0	21.0	3.0	6.0	5.0	45.0	24.0	4.0	3.0	4.0	4.0	5.0	4.0	3.0	4.0	21.0	21.0	21.0	18.0	18.0	18.0	13.0	18.0	30.0	18.0	18.0	18.0	18.0	18.0	18.0	5.0	5.0	1.0	1.0	1.0	1.0	1.0	1.0	1.0	1.0	1.0	2.0	1.0	1.0	3.0	1.0																
TCV10	4.0	3.0	3.0	4.0	10.0	3.0	24.0	20.0	2.0	5.0	4.0	44.0	23.0	3.0	2.0	3.0	3.0	4.0	3.0	2.0	3.0	20.0	20.0	20.0	19.0	19.0	19.0	14.0	19.0	29.0	19.0	19.0	19.0	19.0	19.0	19.0	4.0	4.0	0.0	0.0	0.0	0.0	0.0	0.0	0.0	0.0	0.0	1.0	0.0	0.0	2.0	0.0	1.0															
TCV11	4.0	3.0	3.0	4.0	10.0	3.0	24.0	20.0	2.0	5.0	4.0	44.0	23.0	3.0	2.0	3.0	3.0	4.0	3.0	2.0	3.0	20.0	20.0	20.0	19.0	19.0	19.0	14.0	19.0	29.0	19.0	19.0	19.0	19.0	19.0	19.0	4.0	4.0	0.0	0.0	0.0	0.0	0.0	0.0	0.0	0.0	0.0	1.0	0.0	0.0	2.0	0.0	1.0	0.0														
TCV12	4.0	3.0	3.0	4.0	10.0	3.0	24.0	20.0	2.0	5.0	4.0	44.0	23.0	3.0	2.0	3.0	3.0	4.0	3.0	2.0	3.0	20.0	20.0	20.0	19.0	19.0	19.0	14.0	19.0	29.0	19.0	19.0	19.0	19.0	19.0	19.0	4.0	4.0	0.0	0.0	0.0	0.0	0.0	0.0	0.0	0.0	0.0	1.0	0.0	0.0	2.0	0.0	1.0	0.0	0.0													
TCV13	4.0	3.0	3.0	4.0	10.0	3.0	24.0	20.0	2.0	5.0	4.0	44.0	23.0	3.0	2.0	3.0	3.0	4.0	3.0	2.0	3.0	20.0	20.0	20.0	19.0	19.0	19.0	14.0	19.0	29.0	19.0	19.0	19.0	19.0	19.0	19.0	4.0	4.0	0.0	0.0	0.0	0.0	0.0	0.0	0.0	0.0	0.0	1.0	0.0	0.0	2.0	0.0	1.0	0.0	0.0	0.0												
TCV14	4.0	3.0	3.0	4.0	10.0	3.0	24.0	20.0	2.0	5.0	4.0	44.0	23.0	3.0	2.0	3.0	3.0	4.0	3.0	2.0	3.0	20.0	20.0	20.0	19.0	19.0	19.0	14.0	19.0	29.0	19.0	19.0	19.0	19.0	19.0	19.0	4.0	4.0	0.0	0.0	0.0	0.0	0.0	0.0	0.0	0.0	0.0	1.0	0.0	0.0	2.0	0.0	1.0	0.0	0.0	0.0	0.0											
TCV17	8.0	7.0	7.0	8.0	13.0	7.0	28.0	24.0	6.0	9.0	8.0	48.0	27.0	7.0	6.0	6.0	6.0	7.0	6.0	6.0	7.0	24.0	24.0	24.0	23.0	23.0	23.0	18.0	23.0	33.0	23.0	23.0	23.0	23.0	23.0	23.0	8.0	8.0	4.0	4.0	4.0	4.0	4.0	4.0	4.0	4.0	4.0	5.0	4.0	4.0	6.0	4.0	5.0	4.0	4.0	4.0	4.0	4.0										
TCV2	4.0	3.0	3.0	4.0	10.0	3.0	24.0	20.0	2.0	5.0	4.0	44.0	23.0	3.0	2.0	3.0	3.0	4.0	3.0	2.0	3.0	20.0	20.0	20.0	19.0	19.0	19.0	14.0	19.0	29.0	19.0	19.0	19.0	19.0	19.0	19.0	4.0	4.0	0.0	0.0	0.0	0.0	0.0	0.0	0.0	0.0	0.0	1.0	0.0	0.0	2.0	0.0	1.0	0.0	0.0	0.0	0.0	0.0	4.0									
TCV3	5.0	4.0	4.0	5.0	11.0	4.0	25.0	21.0	3.0	6.0	5.0	45.0	24.0	4.0	3.0	4.0	4.0	5.0	4.0	3.0	4.0	21.0	21.0	21.0	18.0	18.0	18.0	13.0	18.0	30.0	18.0	18.0	18.0	18.0	18.0	18.0	5.0	5.0	1.0	1.0	1.0	1.0	1.0	1.0	1.0	1.0	1.0	2.0	1.0	1.0	3.0	1.0	0.0	1.0	1.0	1.0	1.0	1.0	5.0	1.0								
TCV4	5.0	4.0	4.0	5.0	11.0	4.0	25.0	21.0	3.0	6.0	5.0	45.0	24.0	4.0	3.0	4.0	4.0	5.0	4.0	3.0	4.0	21.0	21.0	21.0	20.0	20.0	20.0	15.0	20.0	30.0	20.0	20.0	20.0	20.0	20.0	20.0	5.0	5.0	1.0	1.0	1.0	1.0	1.0	1.0	1.0	1.0	1.0	2.0	1.0	1.0	3.0	1.0	2.0	1.0	1.0	1.0	1.0	1.0	5.0	1.0	2.0							
TCV5	4.0	3.0	3.0	4.0	10.0	3.0	24.0	20.0	2.0	5.0	4.0	44.0	23.0	3.0	2.0	3.0	3.0	4.0	3.0	2.0	3.0	20.0	20.0	20.0	19.0	19.0	19.0	14.0	19.0	29.0	19.0	19.0	19.0	19.0	19.0	19.0	4.0	4.0	0.0	0.0	0.0	0.0	0.0	0.0	0.0	0.0	0.0	1.0	0.0	0.0	2.0	0.0	1.0	0.0	0.0	0.0	0.0	0.0	4.0	0.0	1.0	1.0						
TCV6	4.0	3.0	3.0	4.0	10.0	3.0	24.0	20.0	2.0	5.0	4.0	44.0	23.0	3.0	2.0	3.0	3.0	4.0	3.0	2.0	3.0	20.0	20.0	20.0	19.0	19.0	19.0	14.0	19.0	29.0	19.0	19.0	19.0	19.0	19.0	19.0	4.0	4.0	0.0	0.0	0.0	0.0	0.0	0.0	0.0	0.0	0.0	1.0	0.0	0.0	2.0	0.0	1.0	0.0	0.0	0.0	0.0	0.0	4.0	0.0	1.0	1.0	0.0					
TCV7	4.0	3.0	3.0	4.0	10.0	3.0	24.0	20.0	2.0	5.0	4.0	44.0	23.0	3.0	2.0	3.0	3.0	4.0	3.0	2.0	3.0	20.0	20.0	20.0	19.0	19.0	19.0	14.0	19.0	29.0	19.0	19.0	19.0	19.0	19.0	19.0	4.0	4.0	0.0	0.0	0.0	0.0	0.0	0.0	0.0	0.0	0.0	1.0	0.0	0.0	2.0	0.0	1.0	0.0	0.0	0.0	0.0	0.0	4.0	0.0	1.0	1.0	0.0	0.0				
TCV8	4.0	3.0	3.0	4.0	10.0	3.0	24.0	20.0	2.0	5.0	4.0	44.0	23.0	3.0	2.0	3.0	3.0	4.0	3.0	2.0	3.0	20.0	20.0	20.0	19.0	19.0	19.0	14.0	19.0	29.0	19.0	19.0	19.0	19.0	19.0	19.0	4.0	4.0	0.0	0.0	0.0	0.0	0.0	0.0	0.0	0.0	0.0	1.0	0.0	0.0	2.0	0.0	1.0	0.0	0.0	0.0	0.0	0.0	4.0	0.0	1.0	1.0	0.0	0.0	0.0			
TCV9	4.0	3.0	3.0	4.0	10.0	3.0	24.0	20.0	2.0	5.0	4.0	44.0	23.0	3.0	2.0	3.0	3.0	4.0	3.0	2.0	3.0	20.0	20.0	20.0	19.0	19.0	19.0	14.0	19.0	29.0	19.0	19.0	19.0	19.0	19.0	19.0	4.0	4.0	0.0	0.0	0.0	0.0	0.0	0.0	0.0	0.0	0.0	1.0	0.0	0.0	2.0	0.0	1.0	0.0	0.0	0.0	0.0	0.0	4.0	0.0	1.0	1.0	0.0	0.0	0.0	0.0		
XE29	26.0	26.0	26.0	27.0	34.0	26.0	6.0	30.0	25.0	29.0	28.0	51.0	33.0	27.0	26.0	26.0	26.0	27.0	26.0	26.0	26.0	30.0	30.0	30.0	31.0	31.0	31.0	32.0	31.0	14.0	31.0	31.0	31.0	31.0	31.0	31.0	28.0	28.0	26.0	26.0	26.0	26.0	26.0	26.0	26.0	26.0	26.0	27.0	26.0	26.0	26.0	26.0	27.0	26.0	26.0	26.0	26.0	26.0	30.0	26.0	27.0	27.0	26.0	26.0	26.0	26.0	26.0	
XE51	22.0	22.0	22.0	23.0	30.0	22.0	2.0	28.0	21.0	25.0	24.0	49.0	31.0	23.0	22.0	22.0	22.0	23.0	22.0	22.0	22.0	28.0	28.0	28.0	29.0	29.0	29.0	28.0	29.0	10.0	29.0	29.0	29.0	29.0	29.0	29.0	24.0	24.0	22.0	22.0	22.0	22.0	22.0	22.0	22.0	22.0	22.0	23.0	22.0	22.0	22.0	22.0	23.0	22.0	22.0	22.0	22.0	22.0	26.0	22.0	23.0	23.0	22.0	22.0	22.0	22.0	22.0	4.0
